# Inferring the Gene Network Underlying the Branching of Tomato Inflorescence

**DOI:** 10.1371/journal.pone.0089689

**Published:** 2014-04-03

**Authors:** Laura Astola, Hans Stigter, Aalt D. J. van Dijk, Raymond van Daelen, Jaap Molenaar

**Affiliations:** 1 Biometris, Wageningen University and Research Centre, Wageningen, The Netherlands; 2 Netherlands Consortium for Systems Biology, Amsterdam, The Netherlands; 3 Keygene N.V., Wageningen, The Netherlands; Leibniz-Institute for Farm Animal Biology (FBN), Germany

## Abstract

The architecture of tomato inflorescence strongly affects flower production and subsequent crop yield. To understand the genetic activities involved, insight into the underlying network of genes that initiate and control the sympodial growth in the tomato is essential. In this paper, we show how the structure of this network can be derived from available data of the expressions of the involved genes. Our approach starts from employing biological expert knowledge to select the most probable gene candidates behind branching behavior. To find how these genes interact, we develop a stepwise procedure for computational inference of the network structure. Our data consists of expression levels from primary shoot meristems, measured at different developmental stages on three different genotypes of tomato. With the network inferred by our algorithm, we can explain the dynamics corresponding to all three genotypes simultaneously, despite their apparent dissimilarities. We also correctly predict the chronological order of expression peaks for the main hubs in the network. Based on the inferred network, using optimal experimental design criteria, we are able to suggest an informative set of experiments for further investigation of the mechanisms underlying branching behavior.

## Introduction

The branching behavior of a tomato (*Solanum lycopersicum*) inflorescence is an important trait for tomato growers and breeders. Depending on the breeding goal, one wants trusses that show no branching at all, a very high level of branching, or just a few branches. The trait is under genetic control and several genes are known to be involved in determining the flowering and structure of the inflorescence. Controlling the shape can therefore be facilitated by proper genetic makeup. Several genes have been identified to be involved in the branching of tomato inflorescence [1], [2]. According to the literature, the following seven genes are essential: S (COMPOUND INFLORESCENCE), J (JOINTLESS), BL (BLIND), AN (ANANTHA), UF (UNIFLORA), FA (FALSIFLORA), and TMF (TERMINATING FLOWER). Most studies focus on a subset of these seven genes, and an overview of how all these genes would fit in a network has not yet been described. As input we use expression data recently published by Park et al. [3]. In their work the genetic basis resulting in different branching behavior of tomato inflorescence in three different genotypes was investigated, namely in a wild-type cultivated tomato, in a mutant, and in a wild species. As illustrated in the paper of Park et al. [3] each of these show different degrees of branching. Based on analysis of the expression data, using the so-called digital differentiation index introduced in [4], they concluded that a high level of branching is driven by the delayed maturation of apical and lateral meristems. In the same spirit, we employ expression data of three distinct tomato genotypes: *S. lycopersicum*, *s* mutant, and *Solanum peruvianum*, measured at different developmental stages in the primary shoot meristems.

Instead of a merely descriptive analysis, we use mathematical modeling to infer the genetic network underlying the branching phenomenon. In general, inferring a regulatory network from given gene expression data is a difficult task. Numerous studies have contributed to this subject in the past, but each of the suggested methods seems to have superior performance only under specific circumstances, whereas none of them is able to claim this in general [5]–[10]. In this article, we are dealing with time series data, which makes information theoretic inference approaches [11] less appropriate. Moreover, our data consists of very limited amount of time points, and therefore methods like dynamic Bayesian networks [12] are not suitable. Rather, a method based on ordinary differential equation (ODE) [13] can potentially capture the dynamics of the developmental network in question without being too detailed to lead to an underdetermined system with a large number of parameters. Inference methods based on systems of ODEs can be divided according to whether they do direct inference using linear regression [14] or fit the network parameters iteratively using optimization algorithms. Our approach belongs to the latter. Further, the inference methods can be divided to approaches that estimate the network parameters and the topology simultaneously by including a penalty term in the objective function [15] and to those that estimate the parameters systematically on different network models [16]. Our method belongs to the latter group, that has the merit that one does not have to come up with a threshold to decide when an parameter is small enough to imply that the corresponding interaction (network edge) does not exist.

Although generic methods have their theoretical appeal as such, when biological prior information is available it is more practical to utilize this information than to infer large amount of unknown parameters “from scratch”. In this paper we rely on literature-based expert knowledge that allows us to generate an initial network that contains the most likely interactions relevant for the inflorescence branching behavior. We want to explore all networks that are in some sense close to our initial network and test whether they are capable to reproduce the data of all three different genotypes. For this, we need two types of criteria: one for comparing networks on their quality and another that determines how to sample the network space. For the former criteria we use the Akaike Final Prediction Error (AFPE) [17]. For the latter, we have developed a procedure, the so-called thickening-thinning-procedure, that provides a heuristic algorithm to navigate in the space of asymmetric adjacency matrices that define the network topology.

The resulting network is not only able to describe the observed expression levels correctly, but can be used to predict how these respond to perturbations. Our model predictions confirm the idea that delayed maturation of meristems is involved in the extreme branching of the *s* mutant. Importantly, our model allows to investigate whether and how strongly this delay depends on the strength of the various interactions in the network. In particular, we find that only very few interactions seem to be responsible for the clear delay between *S. lycopersicum* and *s* mutant. However, the cause of the delay in maturation times between *S. lycopersicum* and *S. peruvianum* seems to be less definitive and can be influenced by several single gene perturbations. Finally, we perform perturbation analysis to select the most influential genes and interactions, to serve as a guideline in designing new experiments.

## Methods

### Determining the network model

We aim at inferring the genetic interactions underlying the branching behavior of tomato inflorescence. For computational modeling, it is useful to conceptualize these interactions in the form of a network graph. The graph consists of nodes that represent the status (expression level) of genes and arrows between them indicate the direction and nature (activation/inhibition) of interaction. We intermittently replace the word “arrow” with “edge”.

In network inference we first need to decide how to describe the dynamics of the network: the mathematical/computational formulation of the processes of genetic interactions in time. How to model a network depends among other things on the nature of the available data. In our case, the point of departure is time series data, where the expression levels of genes are measured at 5 different developmental stages in the shoot meristems [3]. When studying systems (of the size as discussed here) in time, a natural framework for modeling is to use ordinary differential equations (ODE). In Appendix S1 we present the ODE model used throughout this paper. Such a linear ODE model has often been used to get an impression of possible interactions between genes of interest [18]–[20]. It has the advantage that to each interaction, precisely one parameter is attached. We use this model to “probe” for potential interactions between the nodes based on data, i.e., our prime interest is to determine whether an arrow exists and if so, whether it corresponds to activation or inhibition.

### Choosing the potential edges of the network

There is a reasonable amount of literature concerning the genes that are involved in controlling and regulating the inflorescence branching. Typically this information is deduced from the phenotype of single, double, or even triple gene mutations, and from various molecular gene expression studies [21]–[30]. Up to now, the set of interactions between all these genes is poorly understood. In the following we present a summary of all putative interactions (i.e., edges between the genes/nodes). For almost none of the gene-pairs is the interaction actually proven.

The BL (BLIND) and UF (UNIFLORA) genes (so-called boundary genes) are involved in the inflorescence architecture and have a role in the development or initiation of secondary axillary meristems [31]. This is also suggested by the fact that *bl* mutants have less flowers (originating from partially or completely undeveloped, axillary meristems [22]). The expression patterns of BL and UF are largely similar [32] implying a possible connection between UF and BL. As these two genes are active very early in the development, it seems reasonable to expect, that they do not directly interact with genes such as AN (ANANTHA) or S, as these are expressed later on and have a role in determining the fate of the meristems to be formed.

**Figure 1:**
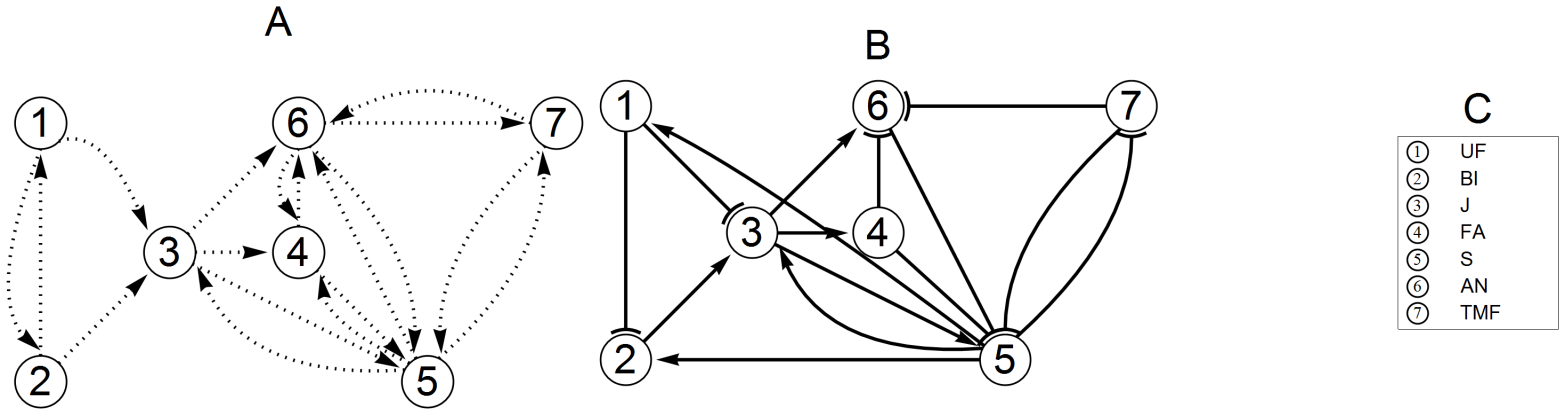
Initial and final networks. The network in panel A shows the set of potential edges to start with. The arrows indicate the direction of interaction. Note that in panel A the arrows may represent activation or inhibition. To avoid cluttering, we have not plotted the arrows for self-regulations, but we do allow them in our model. The final network in panel B was obtained using the network inference procedure that we propose in this paper. In panel C the genes associated with each node are listed.

The genes J (JOINTLESS), S (COMPOUND INFLORESCENCE), AN, and FA (FALSIFLORA) are all expressed at some stage during the inflorescence development. The J-gene has an important role in maintaining the inflorescence status, as mutation of this gene always leads to vegetative growth [23]. Furthermore it is present throughout the inflorescence meristems, but less so in flowers [1], [33]. The J-gene becomes expressed already very early and there are indications that it can potentially interact with S, FA, and AN [1], [23], as well as with the even earlier genes UF or BL. Especially for BL an interaction with J is quite likely, considering the correlation of the expression levels of these genes in *j* mutants in a study on the development of the abscission zone in the pedicel [34]. AN is expressed only in floral buds and most likely only after expression of J and S [35]. Both AN and FA are needed for floral identity [24], [25], [35], as suggested by the fact that their mutants show incompletely developed flowers. Therefore these genes are the latest expressed genes during inflorescence/flower development. We assume that they are not connected to the early genes BL and UF. *S* mutants have highly branched inflorescence, but normal flowers. This was interpreted in [35] as an extension of the indeterminate state resulting in delayed transition to floral meristem. Thus, S gene potentially interacts with other floral decision genes as AN, J, TMF, and FA, but neither with UF nor with BL. The TMF gene may interact with AN and FA but is not likely to be connected to BL or UF, considering the different roles in the development of the inflorescence. Representing all potential edges in a graph, yields the network topology as shown in panel A of Figure 1.

### Modeling the genotypes

The *s* mutant contains a mutation which results in the S-gene being less active than in the wild type [35]. It was first described around 100 years ago as a highly branched variety. We implement this in our computational model by using identical parameter sets for *S. lycopersicum* and *s* mutant, with the following exception: the influence of gene S upon other nodes in the network is mitigated via a multiplicative factor 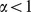 in the equations modeling the *s* mutant. This 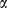 indicates the degree of loss in function of gene S compared to the wild type. As explained in Appendix S1 in detail, we require thus that our model parameters simultaneously predict both genotypes, *S. lycopersicum* and *s* mutant, and that the apparent differences in expression data between the cultivated wild type and its s mutant can be modeled with only one parameter 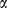. As for the wild species *S. peruvianum*, which is a more distant variety, we expect that the regulatory network is the same in its structure and mechanisms, that is, the interaction mechanisms (activation/inhibition) are the same, but their interaction strengths may differ from that of *S. lycopersicum* and *s* mutant. In terms of mathematical network inference this means that we use the same system of equations for *S. peruvianum* as we obtained for *S. lycopersicum* with identical plus-minus signs but allow the parameter magnitudes to deviate.

### Inferring the network topology

**Figure 2:**
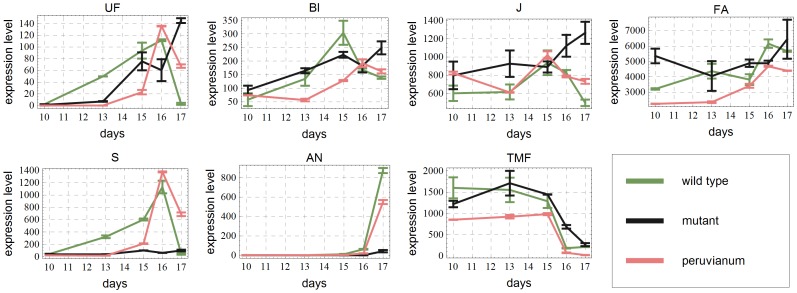
Original data. Expression levels in a.u. for the three genotypes used in this paper as a function of time. At days 10,13,15,16 and 17 data are available. Also standard deviations are given.

The data to be fitted, i.e., the original expression levels for each developmental stage and for each genotype are shown in Figure 2. The main goal of network inference is to find the locations of the actual edges, i.e., the pairs of genes that influence each other. In a computational model, missing a necessary edge in the network will result in a system of equations, whose solutions do not describe the data sufficiently well. On the other hand, a redundant edge (parameter) can be removed from the equations without decreasing the quality of the fit. This insight forms the basis of the inference algorithm explained underneath. For all the details concerning the (pre-processing of) data and the system of differential equations modeling the expression dynamics, we refer the reader to Appendix S1.

#### Thickening-phase

We begin by fitting the initial network (see panel A of Figure 1) to the data of wild type cultivated tomato and mutant. This means that we employ optimization algorithms to find optimal parameters for our model system so that the solution curves follow the data as closely as possible. It is not to be expected that we may fit the data well with this network, since it is based on a-priori knowledge with uncertainties. So, we start “thickening” the network by adding necessary edges to improve the fit. For this, we use a strategy to detect missing edges in the network. In [36], it was shown that in case of small networks with, say, less than 12 nodes, an effective way to pin down the missing edges is to focus on the lack of fit for each gene (node). The idea is that a bad fit for a certain gene often implies that this particular gene is not yet connected to its actual regulator.

To measure the (lack of) fit we use as goodness of fit measure the relative root mean square of the error (RMSE) between data and fit. That is, we measure the difference between data and fit relative to the data values. This means that we do not only fit the highest expression levels well meanwhile ignoring the lower values, but strive to fit each expression data on equal terms. The smaller the RMSE, the better the fit.

To begin with the thickening algorithm, we choose the two nodes, with the largest errors in fit. We connect these two nodes and perform a new fitting. In case the fit is not immediately improved, we systematically try out all edges starting from/ending to these two nodes and choose the edge that reduces the RMSE most.

By repeating this scheme over and over again, we would end up with a fully connected network. To avoid over-fitting, we need a stopping criterion for thickening. For this we apply the Akaike Final Prediction-error Criterion (AFPE) [17], [37]:
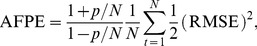
(1)where 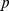 is the number of parameters (edges) and 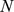 is the number of measurements. Only if the value of AFPE does not increase, we add edges to improve the fit. We remark that, unlike the standard Akaike information criterion (AIC), AFPE takes the number of measurements into account and penalizes the number of parameters less severely. The AFPE is based on solid theory and has the advantage that, in our experience, it leads to a results that better agree with the visual perception of a “good fit”. After the thickening phase, we start eliminating redundant edges by a thinning procedure.

#### Thinning-phase

In the thinning procedure we compute the sensitivity of the RMSE (goodness of fit) with respect to small perturbations in each parameter. If the RMSE hardly changes while we manipulate the values of a parameter (edge), then this edge is not likely to be relevant in the current configuration. The parameter with lowest sensitivity, i.e., the parameter that has practically no effect on the goodness of fit is then deleted from the parameter set. This implies that we delete a network edge and make the network thinner. Again, as a stopping criterion, we use the AFPE criterion introduced above. In case the AFPE criterion for the reduced network is lower than for the original one, we replace the initial network with the reduced network. Note that we are not primarily interested in the parameter values themselves. Rather we want to know their signs, i.e., whether the regulatory action is promotion or inhibition. Therefore, a classical parameter uncertainty analysis is not directly relevant here.

## Results and Discussion

### Necessary and redundant edges

In this section, we discuss the results obtained by applying the thickening-and-thinning procedure, described in the previous section. With the initial network topology (see Figure 1 A), we obtained an insufficient fit, where especially the RMSE for the expression data of UF and BL was relatively large. Therefore, we started thickening the network. As node UF and node BL are already doubly connected to each other, adding an edge between the two was ruled out. Therefore, we systematically connected all remaining nodes one by one first to UF and then to BL, while recording the AFPE values for each fit. Only after adding two new edges to the network: S→UF and S→BL the RMSE dropped to 50% of the original, reducing immediately also the AFPE value.

**Figure 3:**
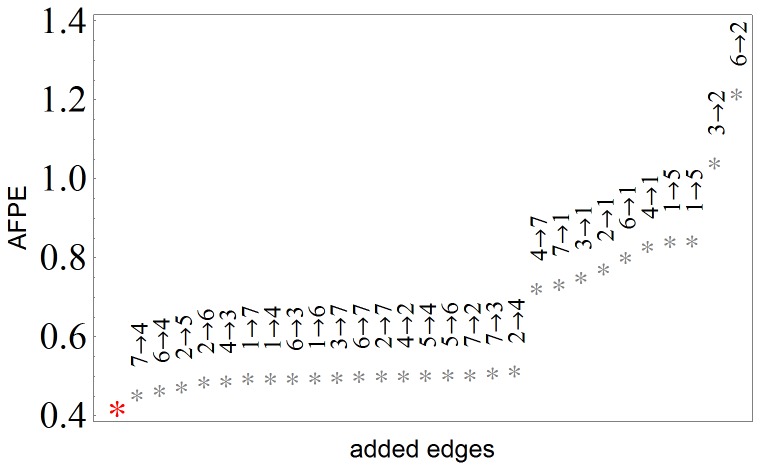
Final prediction errors. The AFPE values obtained during the second thickening phase. The leftmost red star corresponds to the AFPE value of the network after the initial thinning phase. Next to this, are the sorted AFPE-values shown together with the corresponding candidate-edge added to the network for parameter estimation. Since adding any edge, not already present in this network, resulted in larger AFPE than without the addition, further thickening is impossible and we stop the iteration.

At this point, we applied thinning. As a result arrows: BL→UF, AN→TMF, AN→FA, S→FA and S→AN turned out to be redundant and were removed. Then we again switched to the thickening phase. Starting again from the nodes with highest RMSE, we systematically add one edge and discard it in case the AFPE increases. In the end we tried every possible edge that is not yet in the network, but in all cases AFPE increased implying that the fit cannot be improved. The AFPE values corresponding to each addition of an edge is shown in Figure 3.

**Figure 4:**
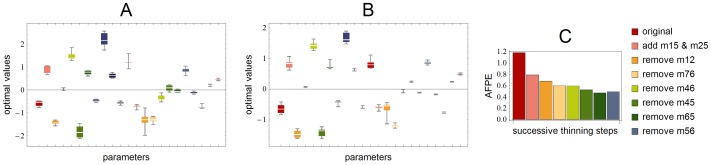
Box-plots of estimated parameters. In panel A is a box-plot of the distribution of the successive optimal parameters during the thinning procedure. The values have consistent signs and narrow range. In panel B are the distributions of successive optimizations using the inferred network structure, starting from different random initial guesses with the Matlab routine lsqnonlin, showing again a narrow range of deviation. In panel C we see the evolution of the AFPE-value during the thickening-thinning procedure. We stopped before the last step, where AFPE increases slightly.

The final fit has thus 2 additional edges and 5 removed edges compared to the original configuration. For the evolution of the AFPE, throughout the thickening and thinning steps, see Figure 4, panel C. Note that throughout the thickening-thinning procedure, we have simultaneously fitted both data, wild type and mutant, which have rather different dynamics, using the same set of parameters with only the special parameter 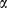 accounting for the differences. The value of 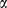 steadily converged to around 0.5, indicating that the influence of the S-gene is 50% weaker in the mutant compared to the wild type. Note that we use global non-constrained optimization without any fixed initial points. Nevertheless the signs of the parameters remained consistent throughout the iteration. For a box-plot of the remaininig optimal parameters during the thinning phase see Figure 4, panel A. As a result, we obtained the minimal network in panel B of Figure 1 that is able to describe the data well. This network contains as many edges as is needed to fit the data, but removing any of them will result in a very poor fit. The algorithm not only unravels which interactions are necessary, but also whether it is a promoting or inhibiting one.

### Predicting the wild species

**Figure 5:**
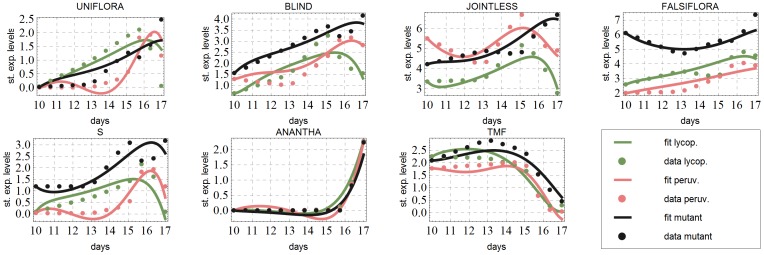
Final fits for all three genotypes. For ease of comparison, we plotted here a subset of the reproduced curves at the same scale using expression levels divided by the standard deviation. Fitting first the data of *S. lycopersicum*, *s* mutant (calibration) and subsequently with the network obtained fitting the data of *S. lycopersicum* (validation).

Using the network inferred via the thickening-thinning procedure explained above, we arrived at a network model that fits both mutant and wild type data. The next question was then whether this network can also predict the data of the more distant variety of tomato, *S. peruvianum*. And if so, are the optimal parameter values significantly different. By fitting the data of *S. peruvianum* with the model inferred on the data of *S. lycopersicum* and *s* mutant we obtained a remarkably good fit. The obtained fits for all three genotypes are given in Figure 5. The parameter values typically varied between 50% to 300% of those from wild type and mutant. Only the parameters 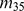 and 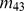 were significantly smaller, corresponding to edges S→J and J→FA in the optimal network for *S. peruvianum*.

### Time delays in expression peaks

**Figure 6:**
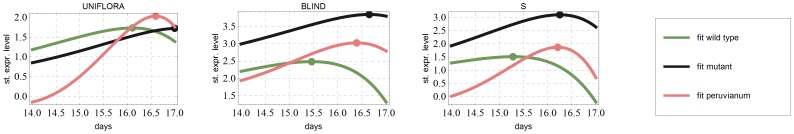
Time delay in expression peaks. The early genes UF and BL as well as the central hub in the network, the S-gene, are plotted, zooming in to the region where the expression levels reach their maximum. Expression levels peak first in the wild type *S. lycopersicum*, secondly in *S. peruvianum* and as last in *s* mutant. The points corresponding to maximal value are represented with dots. This result is in line with the findings of Park et al. on the chronological order of apical meristem maturation in the three genotypes.

Park et al. [3] propose that the difference in branching patterns of cultivated tomato, *s* mutant, and wild species *S. peruvianum*, is due to delayed maturation of meristems. To study this we only need to observe when the expressions of the genes responsible for maturation show a peak in the model prediction. Hence, based on this assumption we can investigate the timings of maturation, presumably influencing the branching behavior, in different tomato variants. To do so, we used the network inferred and given in Figure 1 B and compared the dynamics predicted in each of the three model variants. Indeed, we found the same order of maturation as proposed by Park et al. [3] (Figure 6). In particular, the early genes UF and BL as well as the central hub in the network, S, peak in the following order: first *S. lycopersicum*, then *S. peruvianum*, and finally *s* mutant. This is in agreement with the maturation order of apical meristems in those species. As can be seen in Figure 2, the original data do not contain such ordering between the peaks, indicating that this behavior emerges from the network model and not directly from the data.

To investigate this further, we performed perturbation analysis to see whether it is possible to change the parameter values and maintain a reasonable fit, so that the order in peaking is altered. First we compared the genotypes *S. lycopersicum* and *s* mutant and observed that except for the parameter indicating the regulation J→AN, it is not possible to perturb the parameters to the extent that the expression levels of UF, BL and J would peak earlier in *s* mutant without totally ruining the fit. On the other hand, when comparing the cultivated tomato with the wild species, several parameters, namely S→TMF, FA→AN, FA→S, J→FA, UF→J, S→BL, UF→BL and S→UF could be perturbed so that it results in the alignment/altering of the peak order. This indicates that the later peaking of the most influential genes in *s* mutant compared to the *S. lycopersicum* is a consistent feature of the network. The details of this analysis are given in Appendix S1.

## Conclusions

We have employed a system of linear ODEs to reconstruct the network underlying the branching behavior of tomato inflorescence. As often is the case, the real difficulty lies in the extremely large number of possible network topologies. Combined with the fact that optimization of parameters in ODE systems is rather time consuming, an exhaustive search can easily become intractable. The central question is then how to navigate through the massive space of all possible network graphs. To overcome this problem, we developed a procedure called the thickening-thinning algorithm. With this algorithm we first guarantee that our network can reproduce the data and subsequently we make sure that the network does not contain redundant edges that are not necessary to fit the data.

We used the data for *S. lycopersicum* and its *s* mutant to reconstruct the underlying network topology. Next we showed that the same network topology is also able to fit the data for *S. peruvianum* quite well. This strongly suggests that we have discovered the correct topology. This conclusion is further underpinned by the observation that it also leads to correct predictions of maturation peaks of the influential genes UF, BL, and S. That is, these genes clearly first peak for *S. lycopersicum*, then for *S. peruvianum* and as last for the *s* mutant. This chronological order is in line with the results of Park et al. (2012) who concluded that the delayed maturation (compared to *S. lycopersicum*) of both the apical and lateral meristems is causing the extreme branching in *s* mutant and that for the *S. peruvianum* this delay was present also but only in the apical meristems.

Using the inferred network we could test the consistency of this peaking order via perturbation analysis and found that the delay between the wild type cultivated tomato and *s* mutant is consistent and cannot be easily altered via up-/down-regulations, whereas the delay between wild type cultivated tomato and wild species is much more susceptible to perturbations.

Finally, using a well-established measure of information content in optimal experimental design [38] we were able to select the most important parameters that point towards those genes that give largest effects upon perturbation.

## Supporting Information

Appendix S1 **Supplementary information on the data, the ODE model and perturbation analyses.**
(PDF)

Figure S1 **In this figure the effect of perturbing each network parameter on the peaking time of gene S is illustrated.** In panel A, *S. lycopersicum* and mutant are compared. In panel B *S. lycopersicum* and *S. peruvianum* are compared. In both panels A and B white squares mean: no change in the chronological order by the parameter perturbation. Gray squares indicate that both expression peaks take place within the same hour. Black square means the peaking times of two genotypes have changed in chronological order. In panel C and D the general sensitivity of the fit to parameter perturbations is shown for comparison. A black square means that the residual has grown 100 fold compared to the original residual with the optimal parameters.
(TIFF)

Table S1The Fisher information values in this table are scaled through dividing by the largest value on the diagonal of the Fisher matrix given by formula (2). These results were obtained using the set of parameters that yielded the best fit to the data for both *S. lycopersicum* and *s* mutant. We observe that the parameter with highest FIM is 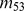, describing the interaction J
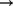
S.(PDF)
