# Supplementary material for: Inferring the Gene Network Underlying the Branching of Tomato Inflorescence
Source: PLoS One. 2014 Apr 3;9(4):e89689. doi: 10.1371/journal.pone.0089689 (PMC3974656; doi:10.1371/journal.pone.0089689)
Supplement: Appendix S1 — Supplementary information on the data, the ODE model and perturbation analyses. (PDF) [file pone.0089689.s001.pdf]

Appendix

Data

Our data is the publicly available annotated and quantified Illumina sequencing data provided by Park et al[3]. It consists of two replicates measured in five different developmental stages in shoot meristems. In our time series analysis, we use the mean of the replicated measurements. We made the explicit assumption that we do not expect substantial fluctuations between the measurements and used linear interpolation between the points. This is necessary to avoid fittings that perfectly fit the actual measurements, but show unwanted oscillations between the data points. From the data, we extracted the measurements of the seven genes: Solyc09g005070.1.1, Solyc11g069030.1.1, Solyc11g010570.1.1, Solyc03g118160.1.1, Solyc02g077390.1.1, Solyc02g081670.1.1, and Solyc09g090180.1.1 (corresponding to UF, BL, J, FA, S, AN, TMF).

Mathematical Model

We try to capture the dynamics of the seven gene network using a model that consists of a system of seven linear ordinary differential equations (ODEs). We remark that using a more complex non-linear model will more than double the number of unknown parameters, which would not be sensible in the light of the currently available amount of data. Here we write down the explicit set of ODEs corresponding to the initial network topology.

In the equations, the variable x_1 corresponds to node 1, variable x_2 to node 2, etc. The nodes correspond to genes as indicated in Figure 1. We recall that an arrow in the network corresponds to a parameter in the ordinary differential equations that represent the system. For example, parameter m_{12} corresponds to an arrow from node 2 to node 1 and so forth. A parameter m_{ii} corresponds to the degradation of variable x_i. The following equations correspond to gene interactions given in the original network topology in panel A of Figure 1:

$$
\begin{aligned}
\dot{x}_1(t) &= m_{11}x_1(t) + m_{12}x_2(t) \\
\dot{x}_2(t) &= m_{21}x_1(t) + m_{22}x_2(t) \\
\dot{x}_3(t) &= m_{31}x_1(t) + m_{32}x_2(t) + m_{33}x_3(t) + \alpha m_{35}x_5(t) \\
\dot{x}_4(t) &= m_{43}x_3(t) + m_{44}x_4(t) + \alpha m_{45}x_5(t) \\
\dot{x}_5(t) &= m_{53}x_3(t) + m_{54}x_4(t) + \alpha m_{55}x_5(t) + m_{56}x_6(t) + m_{57}x_7(t) \\
\dot{x}_6(t) &= m_{63}x_3(t) + m_{64}x_4(t) + \alpha m_{65}x_5(t) + m_{66}x_6(t) + m_{67}x_7(t) \\
\dot{x}_7(t) &= \alpha m_{75}x_5(t) + m_{76}x_6(t) + m_{77}x_7(t)\ ,
\end{aligned}
\tag{1}
$$

where for the *S. lycopersicum*, $\alpha = 1$ and for the s mutant $\alpha < 1$ to present the lower activity of the S-gene. On the other hand, we let the *S. peruvianum* to have its own independent parameters p_{ij} and assume only that the network topology (arrows) and the signs (+ for activation, − for inhibition) of the parameters are equal.

To fit the solutions equations (1) to data, we use the `NMinimize` function of Mathematica, which does global minimization without specified initial points or constraints. `NMinimize` can employ 4 algorithms: Nelder-Mead, simulated annealing, random search and differential evolution, but in our experience, typically the best results were obtained by not specifying the particular method and letting `NMinimize` make the decision internally. In parallel, we did 200 optimizations starting from different initial guesses using the `lsqnonlin` of Matlab, that employs as default the trust-region-reflective algorithm. The distribution of the optimal parameters depends on the value of the accepted residual and as the residual decreases, the parameter distribution converges to a narrow peaked multivariate distribution as can be seen in the panel B of Figure 6.

Perturbation analyses

Fisher information criterion

To investigate which parameters, have maximal influence on the interactions in the system, we did two types of computational perturbation experiments. In designing theoretically optimal experiments, a popular tool is the so-called Fisher information criterion (FIM)[38].

$$\mathrm{FIM} = \int_{t_1}^{t_2} \sum_{ij} \left(\frac{dx_i(t)}{dm_j}\right)^T Q^{-1} \left(\frac{dx_i(t)}{dm_j}\right) dt \ , \tag{2}$$

where t_1 is the first observation time point, t_2 the last time point and matrix Q normally contains the standard deviations of the measurement noise on the diagonal. In our case, we are focusing on the ratios, i.e., relative influences of the parameters and therefore replace the Q with an identity matrix $I_{7\times7}$. Fisher information integrates the total variation in the solution curves $x_i(t)$, resulting from the perturbation of each parameter m_j. This information value is computed at the optimal point $m = m_{opt}$, and it gives information on the local effect of the system, especially about the degree by which a parameter can influence the system across the time. We used the Fisher information criterion to quantitatively compare the amount of observable effects that we are likely to obtain from different experimental set-ups. We have summarized the Fisher information values in Table S1. From this table we conclude that the most informative parameter in this sense is m_{53}, which connects J to S. Actually, almost every significant value is either connected to J or S, suggesting that modifications targeted to these two genes may be most effective.

Effects of parameter perturbation on the peaking order

We investigated, how sensitive the order of expression peaks in time is to changes in parameters. We perturbed each parameter up to ±20 % of its original value. In panels A and B of Figure S7 we illustrate the sensitivity of the order of the expression peaks. Based on our model we can conclude that via modest parameter perturbations such as up- or down-regulation of certain gene in the network it is not possible to alter the delay in the expression peaks (of UF, BL and S) in *s* mutant compared to those in *S. lycopersicum* without at the same time deteriorating the fit. On the other hand, we find that such perturbations can indeed easily affect the delay in the expression peaks of the wild species. As can be seen from the Figure S7, modest perturbation can cause the expression levels of the influential genes in *S. peruvianum* to peak even earlier than in *S. lycopersicum.* The interpretation is that a black square in panel A (B), indicating the theoretical possibility that a parameter perturbation can cause change in peaking order, is in practice not valid if the corresponding square in panel C (D) is also black since then this perturbation has already deteriorated the fit. We found that it is difficult to alter the peaking order of wild type cultivated tomato and *s* mutant, while changing the order between cultivated tomato and wild species is feasible via various perturbations. This indicates that the time delay in the expression levels indeed is a consistent feature discriminating the highly branched *s* mutant phenotype from the wild type, but not so for the wild species.
