# Supplementary figures and images for: Inferring the Gene Network Underlying the Branching of Tomato Inflorescence
Source: PLoS One. 2014 Apr 3;9(4):e89689. doi: 10.1371/journal.pone.0089689 (PMC3974656; doi:10.1371/journal.pone.0089689)

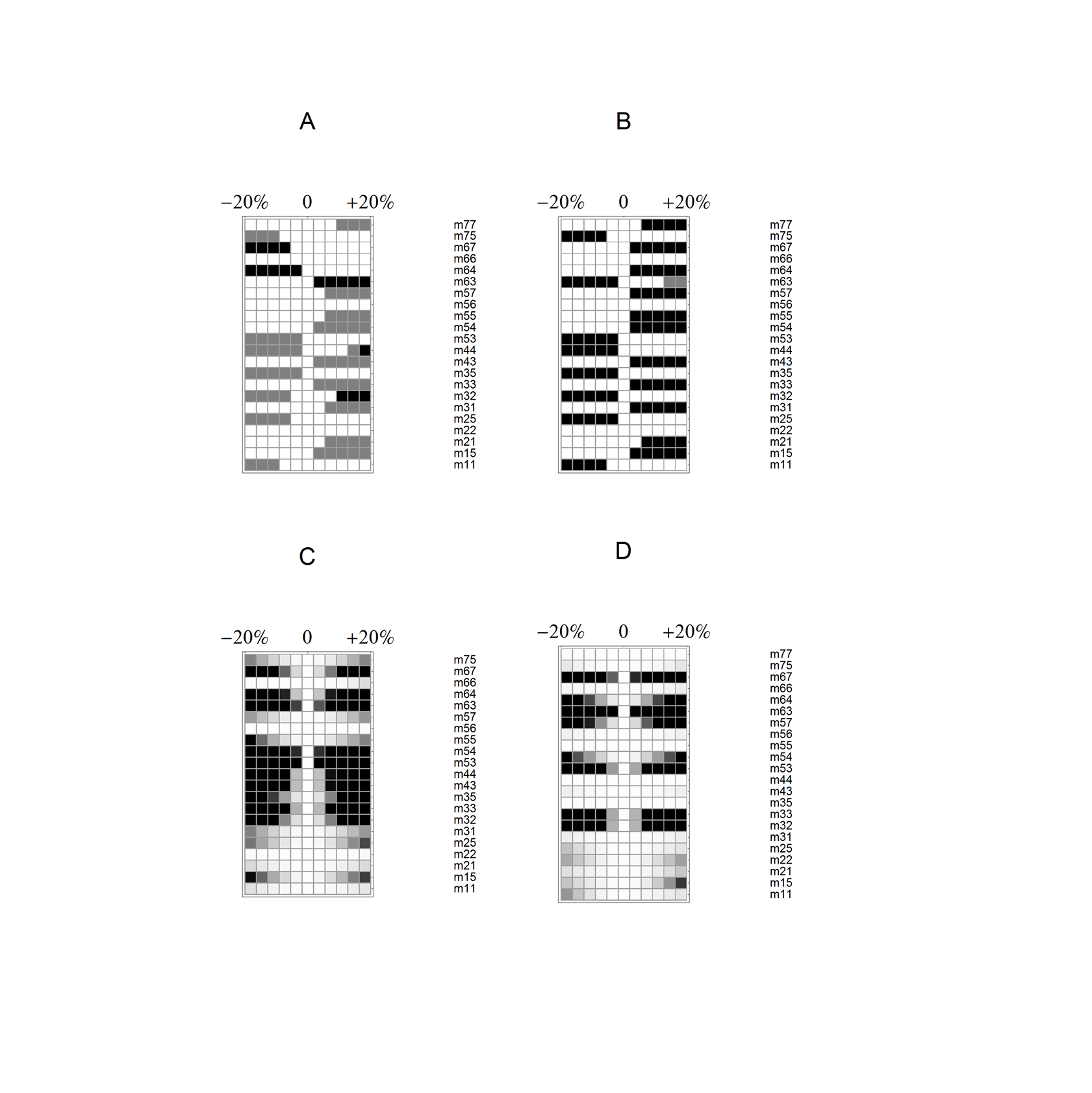

Supplement: Figure S1 — In this figure the effect of perturbing each network parameter on the peaking time of gene S is illustrated. In panel A, S. lycopersicum and mutant are compared. In panel B S. lycopersicum and S. peruvianum are compared. In both panels A and B white squares mean: no change in the chronological order by the parameter perturbation. Gray squares indicate that both expression peaks take place within the same hour. Black square means the peaking times of two genotypes have changed in chronological order. In panel C and D the general sensitivity of the fit to parameter perturbations is shown for comparison. A black square means that the residual has grown 100 fold compared to the original residual with the optimal parameters. (TIFF) [file pone.0089689.s002.tiff]
