# Supplementary material for: Inferring the Gene Network Underlying the Branching of Tomato Inflorescence
Source: PLoS One. 2014 Apr 3;9(4):e89689. doi: 10.1371/journal.pone.0089689 (PMC3974656; doi:10.1371/journal.pone.0089689)
Supplement: Table S1 — The Fisher information values in this table are scaled through dividing by the largest value on the diagonal of the Fisher matrix given by formula (2). These results were obtained using the set of parameters that yielded the best fit to the data for both S. lycopersicum and s mutant. We observe that the parameter with highest FIM is , describing the interaction JS. (PDF) [file pone.0089689.s003.pdf]

parameter	m11	m15	m21	m22	m25	m31	m32	m33	m35	m43	m44
Fisher value	0.00	0.02	0.00	0.00	0.02	0.01	0.07	0.18	0.06	0.15	0.15
parameter	m53	m54	m55	m56	m57	m63	m64	m66	m67	m75	m77
Fisher value	1.00	0.51	0.01	0.00	0.01	0.39	0.11	0.00	0.08	0.01	0.02
